# Mosquito Acetylcholinesterase as a Target for Novel Phenyl-Substituted Carbamates

**DOI:** 10.3390/ijerph16091500

**Published:** 2019-04-28

**Authors:** James M. Mutunga, Ming Ma, Qiao-Hong Chen, Joshua A. Hartsel, Dawn M. Wong, Sha Ding, Max Totrov, Paul R. Carlier, Jeffrey R. Bloomquist

**Affiliations:** 1 Emerging Pathogens Institute, Entomology and Nematology Department, University of Florida, Gainesville, FL 32610, USA; James.Mutunga@usamru-k.org; 2 Department of Chemistry, Virginia Polytechnic Institute and State University, Blacksburg, VA 24061, USA; gym7981@gmail.com (M.M.); qchen@csufresno.edu (Q.-H.C.); jhartsel@vt.edu (J.A.H.); dawnwong@vt.edu (D.M.W.); sding@vt.edu (S.D.); pcarlier@vt.edu (P.R.C.); 3 Molsoft LLC, 11199 Sorrento Valley Road, S209 San Diego, CA 92121, USA; max@molsoft.com

**Keywords:** *Anopheles gambiae*, anticholinesterase, insecticide, toxicity

## Abstract

New insecticides are needed for control of disease-vectoring mosquitoes and this research evaluates the activity of new carbamate acetylcholinesterase (AChE) inhibitors. Biochemical and toxicological characterization of carbamates based on the parent structure of terbam, 3-*tert*-butylphenyl methylcarbamate, was performed. In vitro enzyme inhibition selectivity (*Anopheles gambiae* versus human) was assessed by the Ellman assay, as well as the lethality to whole insects by the World Health Organization (WHO) paper contact assay. Bromination at the phenyl C6 position increased inhibitory potency to both AChEs, whereas a 6-iodo substituent led to loss of potency, and both halogenations caused a significant reduction of mosquitocidal activity. Similarly, installation of a hexyl substituent at C6 drastically reduced inhibition of *Ag*AChE, but showed a smaller reduction in the inhibition of hAChE. A series of 4-carboxamido analogs of the parent compound gave reduced activity against *Ag*AChE and generally showed more activity against hAChE than *Ag*AChE. Replacement of the 3-*t*-buyl group with CF_3_ resulted in poor anticholinesterase activity, but this compound did have measurable mosquitocidal activity. A series of methyl- and fluoro- analogs of 3-trialkylsilyl compounds were also synthesized, but unfortunately resulted in disappointing activity. Finally, a series of sulfenylated proinsecticides showed poor paper contact toxicity, but one of them had topical activity against adult female *Anopheles gambiae*. Overall, the analogs prepared here contributed to a better understanding of carbamate structure–activity relationships (SAR), but no new significant leads were generated.

## 1. Introduction

The use of commercial carbamate insecticides dates back to the late 1950s, and numerous efforts have been made to improve their toxicity to insects. Despite the wide-scale use of carbamates for crop and urban pest control [1], only propoxur and bendiocarb are authorized by the World Health Organization (WHO) for indoor residual spraying of mosquitoes for malaria control [2]. A major concern for deploying carbamates in or near human dwellings is non-target toxicity, and two primary factors determine the selectivity of a carbamate: selective detoxification in vertebrate systems and selective AChE inhibition in insects [3]. Current advances in proteomics and genomics, the availability of both the *Anopheles gambiae* and human AChE sequences, and advanced tools of computational biology enable fine-scale ligand docking, and virtual ligand library screening aid in the design of new AChE inhibitors.

This study describes our continuing efforts to improve carbamate insecticides for mosquito control, building on our previous work with phenyl-substituted analogs [4,5,6,7]. Structural modifications included extending the side-chain branching of alkyl or silyl substituents to explore the effects of spatial and structural complementarity of compounds to the ammonium group of acetylcholine (ACh), since Kolbezen et al. [8] suggested that the structural complementarity of the 3-*tert*-butyl group of terbam **1a** to ACh explained its high potency with bovine AChE. Ring halogenation investigated not only the size of the halogen substituent, but also the preferred placement on the molecule. Carbamate side-chains are conventionally thought to be major detoxication sites [9], and halogenation at these sites typically confers protection against hydroxylation by P450 mono-oxygenases [10]. Inclusion of thioalkyl substituents sought to explore not only the ACh structural complementarity of these analogs, but also the possible bioactivation of sulfur to more toxic sulfoxide and sulfone derivatives, in vivo. These data help to better understand the interactions of carbamates with insect and human AChE and inform the future design of a pharmacophore with superior insect toxicity and selectivity.

## 2. Materials and Methods

### 2.1. Preparation of Carbamates

Carbamates **1a**, **3a**, **4a**, and **5a** were prepared as previously described by Hartsel et al. [5] and the synthesis of other experimental compounds is described below.

4-bromo-3-(tert-butyl)phenyl methylcarbamate (**1b**): 3-t-butylphenol (3 g) was dissolved in 10% NaOH (10 mL), and iodine (5.6 g, 1.1 equivalent) and sodium iodide (5.6 g, 1.5 equivalent) were added to afford 5-(t-butyl)-2-iodophenol (4.96 g, 90% yield). Treatment with bromine in CH_2_Cl_2_ afforded 4-bromo-5-t-butyl-2-iodophenol; refluxing in N-methylmorpholine for 16 h, followed by column chromatography afforded 4-bromo-3-t-butylphenol in 50% yield over two steps. This compound (50 mg) was dissolved in dry tetrahydrofuran (THF) (2 mL), and potassium t-butoxide (0.29 mL, 1 M in THF) was added followed by methylcarbamoyl chloride (2 equiv). After 1 h, aqueous workup and column chromatography afforded **1b** as a pale semi-solid (43 mg, 69%). ^1^H NMR (400 MHz, CDCl_3_) δ 7.53 (d, *J* = 10.0 Hz, 1H), 7.19 (s, 1H), 6.85 (d, *J* = 10.0 Hz, 1H), 4.99 (br s, 1H), 2.95 (d, *J* = 3.0 Hz, 0.15H, minor carbamate rotamer), 2.88 (d, *J* = 3.0 Hz, 2.85H, major carbamate rotamer), 1.50 (s, 9H); ^13^C NMR (101 MHz, CDCl_3_) δ 154.9, 150.2, 149.2, 136.2, 121.6, 120.7, 118.4, 36.7, 29.5, and 27.8.

2-bromo-5-(tert-butyl)phenyl methylcarbamate (**1d**): 3-t-butylphenol (3 g) was dissolved in dichloromethane (30 mL) and bromine (1.1 mL, 1.1 equivalent) was added dropwise. After stirring for 1 h, aqueous workup and concentration in vacuo afforded 2-bromo-5-t-butylphenol in quantitative yield. This phenol was treated as above for **1b** to afford **1d** as pale semi-solid (70% yield). ^1^H NMR (400 MHz, CDCl_3_) δ 7.44 (d, *J* = 8.4 Hz, 1H), 7.16 (d, *J* = 2.0 Hz, 1H), 7.08 (dd, *J* = 8.4, 2.0 Hz, 1H), 5.05 (br s, 1H), 3.00 (d, *J* = 4.8 Hz, 0.15H, minor carbamate rotamer), 2.89 (d, *J* = 4.8 Hz, 2.85H, major carbamate rotamer), 1.26 (s, 9H); ^13^C NMR (101 MHz, CDCl_3_) δ 154.3, 152.1, 147.9, 132.4, 124.3, 121.3, 113.0, 34.7, 31.1, and 27.8.

5-(tert-butyl)-2-iodophenyl methylcarbamate (**1e**): 5-(tert-butyl)-2-iodophenol (described above in synthesis of 1b) was then dissolved in dry THF (10 mL), treated with NaH (60% dispersion in mineral oil, 1.6 equivalent), and methylcarbamoyl chloride (2.5 equiv). After 1 h aqueous workup and column chromatography afforded **1e** as a pale semi-solid (134 mg 50%). ^1^H NMR (400 MHz, CDCl_3_) δ 7.68 (d, *J* = 8.3 Hz, 1H), 7.17(s, 1H), 6.97 (d, *J* = 8.3 Hz, 1H), 5.19 (br s, 1H), 3.01 (br s, 0.15H, minor carbamate rotamer), 2.89 (br s, 2.85H, major carbamate rotamer), 1.28 (s, 9H); ^13^C NMR (101 MHz, CDCl_3_) δ 154.3, 153.3, 150.8, 138.4, 124.6, 120.6, 86.7, 34.6, 31.1, and 27.8.

5-(tert-butyl)-2-hexylphenyl methylcarbamate (**1f**): The requisite phenol was prepared from 5-(t-butyl)-2-iodophenol (see above) in a four-step sequence (62% overall yield) by (i) acetylation, (ii) Sonogashira coupling with 1-hexyne, catalytic hydrogenation, and deacetylation. This phenol was then reacted with NaH and methylcarbamoyl chloride according to the procedure for **1e**, to afford **1f** as a pale semi-solid (110 mg, 58%). ^1^H NMR (400 MHz, CDCl_3_) δ 7.23–7.13 (m, 2H), 7.06 (s, 1H) 5.01 (br s, 1H), 2.94 (d, *J* = 4.8 Hz, 0.15 H, minor carbamate rotamer) 2.88 (d, *J* = 4.8 Hz, 2.85H, major carbamate rotamer), 2.50 (t, *J* = 7.7 Hz, 2H), 1.61–1.52 (m, 2H), 1.36–1.23 (m, 15H), 0.88 (t, *J* = 6.8 Hz, 1H); ^13^C NMR (101 MHz, CDCl_3_) δ 155.4, 150.0, 148.7, 131.8, 129.4, 122.5, 119.4, 34.4, 31.6, 31.2, 29.9, 29.6, 29.2, 27.7, 22.5, and 14.1.

4-(allylcarbamoyl)-3-(tert-butyl)phenyl methylcarbamate (**1h**): 4-bromo-3-t-butylphenol (above in **1b**) was converted to the triiisopropylsilyl derivative (TIPS-Cl, imidazole, DMF), dissolved in diethyl ether, metalated with t-BuLi and trapped with ethylchloroformate, and then hydrolyzed (KOH, aq THF; HCl aq). Treatment with TBS-Cl and imidazole in dichloromethane afford 2-(t-butyl)-4-t-butyldimethylsilyloxybenzoic acid in 57% yield over four steps. Treatment with oxalyl chloride, followed by allyl amine afforded the allyl amide. Deprotection with HF/pyridine, deprotonation with potassium t-butoxide and acylation with methylcarbamoyl chloride afforded **1h** as a pale oil (46% over three steps). ^1^H NMR (400 MHz, CDCl_3_): Note: chiral axis C4-C(O) renders the four CH_2_ protons diastereotopic δ 7.22 (d, *J* = 9.0 Hz, 1H), 7.15 (s, 1H), 6.88 (d, *J* = 9.0 Hz, 1H) 5.88 (ddt, *J* = 15.0, 10.0, 6.2 Hz, 1H), 5.81 (br s, 1H), 5.25 (dd, *J* = 15.0, 2.0 Hz, 1H), 5.22 (dd, *J* = 10.0, 2.0 Hz, 1H), 5.20 (br s, 1H), 4.00 (t, *J* = 6.2 Hz, 2H), 2.94 (d, *J* = 4.0 Hz, 0.15H, minor carbamate rotamer), 2.82 (d, *J* = 4.0 Hz, 2.85H, major carbamate rotamer), 1.45 (s, 9 H); ^13^C NMR (101 MHz, CDCl_3_) δ 172.3, 155.2, 151.6, 149.7, 133.7, 133.2, 129.4, 120.6, 118.8, 117.2, 42.5, 36.4, 31.4, and 27.8.

3-(tert-butyl)-4-(methylcarbamoyl)phenyl methylcarbamate (**1i**): 2-(t-butyl)-4-t-butyldimethylsilyloxybenzoic acid (from **1b** above) was treated with oxalyl chloride, followed by methylamine. Deprotection with HF/pyridine, deprotonation with potassium t-butoxide and acylation with methylcarbamoyl chloride afforded **1i** as a pale oil (42% over three steps). ^1^H NMR (400 MHz, CDCl_3_): Note: chiral axis C4-C(O) renders the four CH_2_ protons diastereotopic δ 7.22-7.13 (m, 2H), 6.89 (d, *J* = 9.0 Hz, 1H), 5.77 (br s, 1H), 5.18 (br s, 1H), 2.93 (d, *J* = 5.0 Hz, 3H), 2.85 (d, *J* = 4.4 Hz, 3H), 1.37 (s, 9H); ^13^C NMR (101 MHz, CDCl_3_) δ 173.2, 155.2, 151.5, 149.6, 133.9, 129.3, 120.5, 118.8, 36.3, 31.3, 27.8, and 26.9.

3-(tert-butyl)-4-(isobutylcarbamoyl)phenyl methylcarbamate (**1j**): 2-(tert-butyl)-4-t-butyldimethylsilyloxybenzoic acid (from **1b** above) was treated with oxalyl chloride, followed by isobutylamine. Deprotection with HF/pyridine, deprotonation with potassium t-butoxide and acylation with methylcarbamoyl chloride afforded **1j** as a pale oil (28% over three steps). ^1^H NMR (400 MHz, CDCl_3_): Note: chiral axis C4-C(O) renders the four CH_2_ protons diastereotopic δ 7.22 (d, *J* = 8.3 Hz, 1H), 7.18 (s, 1H), 6.95 (d, *J* = 8.3 Hz, 1H), 5.80 (br s, 1H), 5.19 (br s, 1H), 3.23 (dd, *J* = 6.2, 5.8 Hz, 2H), 2.85 (d, *J* = 3.8 Hz, 0.15H, minor carbamate rotamer), 2.79 (d, *J* = 3.8 Hz, 2.85H, major carbamate rotamer), 1.90 (t sep, *J* = 6.8, 6.2 Hz, 1H), 1.44 (s, 9H), 0.92 (d, *J* = 6.8 Hz, 6H); ^13^C NMR (101 MHz, CDCl_3_) δ 172.6, 155.2, 151.5, 149.6, 134.1, 129.4, 120.5, 118.8, 47.6, 36.3, 31.3, 28.3, 27.8, and 20.3.

3-(tert-butyl)-4-(diethylcarbamoyl)phenyl methylcarbamate (**1k**): 2-(t-butyl)-4-t-butyldimethylsilyloxybenzoic acid (from **1b** above) was treated with oxalyl chloride, followed by diethylamine. Deprotection with HF/pyridine, deprotonation with potassium t-butoxide and acylation with methylcarbamoyl chloride afforded **1k** as a pale oil (29% over three steps). ^1^H NMR (400 MHz, CDCl_3_): Note: chiral axis C4-C(O) renders the four CH_2_ protons diastereotopic δ 7.20 (s, 1H), 6.98 (d, *J* = 8.0 Hz, 1H), 6.83 (d, *J* = 8.0 Hz, 1H), 5.05 (br s, 1H), 3.72 (dq, *J* = 15.2, 6.2 Hz, 1H), 3.28 (dq, *J* = 15.6, 6.2 Hz, 1H), 3.20 (dq, *J* = 15.2, 6.2 Hz, 1H), 3.01 (dq, *J* = 15.6, 6.2 Hz, 1H), 2.95 (d, *J* = 4.0 Hz, 0.15 H, minor carbamate rotamer), 2.78 (d, *J* = 4.0 Hz, 2.85H, major carbamate rotamer), 1.29 (s, 9H), 1.23 (dd, *J* = 6.2, 6.2 Hz, 3H), 1.02 (dd, *J* = 6.2, 6.2 Hz, 3H); ^13^C NMR (101 MHz, CDCl_3_) δ 172.3, 154.6, 150.8, 147.0, 133.5, 127.7, 120.9, 118.1, 44.2, 38.6, 36.1, 32.0, 27.5, 13.8, and 12.7.

3-(tert-butyl)-4-(ethyl(methyl)carbamoyl)phenyl methylcarbamate (**1l**): 2-(t-butyl)-4-t-butyldimethylsilyloxybenzoic acid (from **1b** above) was treated with oxalyl chloride, followed by ethylmethylamine. Deprotection with HF/pyridine, deprotonation with potassium t-butoxide and acylation with methylcarbamoyl chloride afforded **1l** as a pale oil (59% over three steps). ^1^H NMR (400 MHz, CDCl_3_): Note: chiral axis C4-C(O) renders the four CH_2_ protons diastereotopic δ 7.13 (d, *J* = 1.8 Hz, 1H), 7.02 (dd, *J* = 8.2, 1.8 Hz, 1H), 6.87 (d, *J* = 8.2 Hz, 1H), 5.32 (br s, 1H), 3.69 (dt, *J* = 15.2, 6.8 Hz, 0.5 H), 3.48 (dt, *J* = 15.2, 6.8 Hz, 0.5 H), 3.25 (dt, *J* = 15.2, 6.8 Hz, 0.5 H), 3.05 (s, 1.5 H), 2.98 (dt, *J* = 15.2, 6.8 Hz, 0.5 H), 2.97 (d, *J* = 3.8 Hz, 0.15 H, minor carbamate rotamer), 2.82 (d, *J* = 3.8 Hz, 2.85 H, major carbamate rotamer), 2.75 (s, 1.5 H), 1.32 (s, 9 H), 1.20 (dd, *J* = 6.8, 6.8 Hz, 1.5 H), 0.97 (dd, *J* = 6.8, 6.8 Hz, 1.5 H); ^13^C NMR (101 MHz, CDCl_3_) δ 176.0, 175.0, 155.3, 151.3, 151.2, 148.1, 148.0, 132.6, 131.9, 128.5, 127.8, 120.9, 119.1, 118.9, 46.0, 41.8, 36.9, 36.4, 31.7, 31.2, 27.7, 13.0, and 11.4 (two equally populated rotamers (ethymethylamide); 24 of a possible 28 resonances seen).

5-(tert-butyl)-2-(methylthio)phenyl methylcarbamate (**1m**): 3-t-butylphenol was treated with chlorosulfonic acid (10 equivalent) in dichloromethane for 4 h, and then poured into ice. Extractive workup, reduction with stannous chloride in acetic acid (18 h), and column chromatography afforded 6,6’-disulfanediylbis(3-(t-butyl)phenol). Reduction with sodium borohydride in THF gave 5-(t-butyl)-2-mercaptophenol in 64% yield over three steps. Treatment with methyl iodide (1.1 equiv) and sodium bicarbonate in DMF, followed by deprotonation with potassium t-butoxide in THF and acylation with methylcarbamoyl chloride gave **1m** as a pale semi-solid in 37% yield over two steps. ^1^H NMR (400 MHz, CDCl_3_) δ 7.23 (dd, *J* = 6.6, 1.6 Hz, 1H), 7.19 (d, *J* = 6.6 Hz, 1H), 7.12 (d, *J* = 1.6 Hz, 1H), 5.04 (br s, 1H), 3.07 (d, *J* = 4.0 Hz, 0.15 H, minor carbamate rotamer), 2.91 (d, *J* = 4.0 Hz, 2.85 H, major carbamate rotamer), 2.42 (s, 3H), 1.30 (s, 9H); ^13^C NMR (101 MHz, CDCl_3_) δ 154.9, 150.1, 148.3, 128.1, 127.3, 123.5, 120.2, 34.6, 31.4, 28.0, 15.7.

5-(tert-butyl)-2-(isobutylthio)phenyl methylcarbamate (**1n**): 5-(t-butyl)-2-mercaptophenol (from **1m** above) was treated with isobutyl iodide (2.9 equivalents) and sodium bicarbonate in DMF at 55 °C for 6 h, followed by deprotonation with potassium t-butoxide in THF and acylation with methylcarbamoyl chloride to gave **1n** as a pale semi-solid in 63% yield over two steps. ^1^H NMR (400 MHz, CDCl_3_) δ 7.27 (d, *J* = 6.6 Hz, 1H), 7.18 (d, *J* = 6.6, 1.6 Hz, 1H), 7.12 (d, *J* = 1.6 Hz, 1H), 5.04 (br s, 1H), 3.02, (d, *J* = 3.9 Hz, 0.15H, minor carbamate rotamer), 2.92 (d, *J* = 3.9 Hz, 2.85H, major carbamate rotamer), 2.73 (d, *J* = 5.5 Hz, 2H), 1.81 (nonet (9-let), *J* = 5.5 Hz, 1H), 1.29 (s, 9H), 1.02 (*J* = 5.3 Hz, 6H); ^13^C NMR (101 MHz, CDCl_3_) δ 155.0, 150.9, 149.5, 130.1, 127.0, 123.4, 120.3, 42.5, 34.7, 31.3, 28.4, 28.0, 22.2.

5-(tert-butyl)-2-(isopropylthio)phenyl methylcarbamate (**1o**): 5-(t-butyl)-2-mercaptophenol (from **1m** above) was treated with isopropyl iodide (3.0 equiv) and sodium bicarbonate in DMF at 55 °C for 6 h, followed by deprotonation with potassium t-butoxide in THF and acylation with methylcarbamoyl chloride to give **1o** as a pale semi-solid in 60% yield over two steps. ^1^H NMR (400 MHz, CDCl_3_) δ 7.36 (d, *J* = 6.6 Hz, 1H), 7.19 (dd, *J* = 6.6, 1.6 Hz, 1H), 7.14 (d, *J* = 1.6 Hz, 1H), 5.03 (br s, 1H), 3.33 (sept, *J* = 5.3 Hz, 1H), 3.02 (d, *J* = 3.9 Hz, 0.15H, minor carbamate rotamer), 2.92 (d, *J* = 3.9 Hz, 2.85H, major carbamate rotamer), 1.30 (s, 9H), 1.27 (d, *J* = 5.3 Hz, 6H); ^13^C NMR (101 MHz, CDCl_3_) δ 155.1, 152.1, 150.7, 133.2, 125.3, 134.3, 120.4, 37.9, 34.8, 31.3, 28.0, and 23.4.

3-(trifluoromethyl)phenyl methylcarbamate (**2a**): 3-trifluoromethylphenol (1.09 g) was deprotonated with potassium t-butoxide in THF and treated with methylcarbamoyl chloride to give **2a** as a pale semi-solid (1.16 g, 81% yield). ^1^H NMR (400 MHz, CDCl_3_) δ 7.47-7.44 (m, 2H), 7.40 (br s, 1H), 7.33-7.31 (m, 1H), 5.17 (br s, 1H), 2.95 (d, *J* = 4.0 Hz, 0.15H, minor carbamate rotamer), 2.88 (d, *J* = 4.0 Hz, 2.85H, major carbamate rotamer); ^13^C NMR (101 MHz, CDCl_3_) δ 154.8, 151.3, 132.3, 129.9, 125.3, 123.3 (q, ^1^J_CF_ = 238 Hz), 122.1, 118.9, and 27.8.

4-methyl-3-(trimethylsilyl)phenyl methylcarbamate (**3c**): 3-bromo-4-methylphenol (0.70 g) was dissolved in dry THF (5 mL), cooled to -78 °C, treated with BuLi (3.4 mL, 2.5 M in hexanes, 2.2 equivalent) and trimethylsilyl chloride (1.2 mL, 1.1 g, 2.6 equivalent). After stirring for 30 min the reaction was allowed to warm to 25 °C and the reaction was quenched with aqueous HCl. Extractive workup and column chromatograph afforded 4-methyl-3-trimethylsilylphenol as a colorless oil (630 mg, 93%). Applying the procedure above, the phenol (630 mg) was converted to the methyl carbamate, affording **3c** as a yellow oil (591 mg, 71% yield). ^1^H NMR (400 MHz, CDCl_3_) δ 7.12 (s, 1H), 7.11 (d, *J* = 9.6 Hz, 1H), 6.98 (d, *J* = 9.6 Hz, 1H), 4.97 (br s, 1H), 2.93 (d, *J* = 4.0 Hz, 0.15H, minor carbamate rotamer), 2.85 (d, *J* = 4.0 Hz, 2.85H, major carbamate rotamer), 2.40 (s, 3H), 0.30 (s, 9H); ^13^C NMR (101 MHz, CDCl_3_) δ 155.8, 148.7, 140.6, 140.1, 130.8, 127.1, 122.4, 27.9, 22.5, and −0.13.

3-fluoro-5-(trimethylsilyl)phenyl methylcarbamate (**3g**): 3-bromo-5-fluorophenol (0.19 g) was dissolved in dry THF (9 mL), cooled to −78 °C, treated with BuLi (1.4 mL, 2.5 M in hexanes, 3.3 equivalent) and trimethylsilyl chloride (0.45 mL, 0.39 g, 3.6 equivalent). After stirring for 30 min the reaction was allowed to warm to 25 °C and the reaction was quenched with aqueous HCl. Extractive workup and column chromatograph afforded 5-fluoro-3-trimethylsilylphenol as a colorless oil (66 mg, 66%). Applying the procedure above, the phenol (66 mg) was converted to the methyl carbamate, affording **3g** as a yellow oil (78 mg, 92% yield). ^1^H NMR (400 MHz, CDCl_3_) δ 7.08-6.99 (m, 2H), 6.87 (ddd, *J* = 9.6, 2.3, 2.1 Hz, 1H), 5.12 (br s, 1H), 2.92 (d, *J* = 4.7 Hz, 0.15H, minor carbamate rotamer), 2.87 (d, *J* = 4.7 Hz, 2.85H, major carbamate rotamer), 0.25 (s, 9H); ^13^C NMR (101 MHz, CDCl_3_) δ 162.5 (d, ^1^J_CF_ = 248.4 Hz) 154.9, 151.5, 144.2 (d, ^3^J_CF_ = 4.7 Hz), 121.6 (d, ^3^J_CF_ = 2.9 Hz), 116.5 (d, ^2^J_CF_ = 18.2 Hz), 110.0 (d, ^2^J_CF_ = 24.1 Hz), 27.7, and −1.4.

3-(ethyldimethylsilyl)-4-methylphenyl methylcarbamate (**4c**): 3-bromo-4-methylphenol (0.70 g) was dissolved in dry THF (5 mL), cooled to −78 °C, treated with BuLi (3.4 mL, 2.5 M in hexanes, 2.2 equivalent) and ethyldimethylsilyl chloride (1.36 mL, 1.19 g, 2.6 equivalent). After stirring for 30 min, the reaction was allowed to warm to 25 °C and the reaction was quenched with aqueous HCl. Extractive workup and column chromatograph afforded 4-methyl-3-trimethylsilylphenol as a colorless oil (665 mg, 91%). Applying the procedure above, the phenol (419 mg) was converted to the methyl carbamate, affording **4c** as a yellow oil (419 mg, 77% yield). ^1^H NMR (400 MHz, CDCl_3_) δ 7.13 (s, 1H), 7.11 (d, *J* = 9.0 Hz, 1H), 6.99 (d, *J* = 9.0 Hz, 1H), 5.05 (br s), 2.90 (d, *J* = 4.9 Hz, 0.15H, minor carbamate rotamer), 2.83 (d, *J* = 4.9 Hz, 2.85H, major carbamate rotamer), 2.40 (s, 3H), 0.93 (t, *J* = 8.0 Hz, 3H), 0.79 (q, *J* = 8.0 Hz, 2H), 0.28 (s, 6H); ^13^C NMR (101 MHz, CDCl_3_) δ 155.9, 148.7, 140.7, 139.2, 130.8, 127.5, 122.3, 27.9, 22.5, 7.75, 7.74, and −2.35.

3-(ethyldimethylsilyl)-5-fluorophenyl methylcarbamate (**4g**): 3-bromo-5-fluorophenol (0.27 g) was dissolved in dry THF (9 mL), cooled to −78 Ç, treated with BuLi (2.1 mL, 2.5 M in hexanes, 3.5 equivalent) and ethyldimethylsilyl chloride (0.80 mL, 0.70 g, 4.0 equivalent). After stirring for 30 min the reaction was allowed to warm to 25 °C and the reaction was quenched with aqueous HCl. Extractive workup and column chromatograph afforded 3-ethyldimethylsilyl-5-fluorophenol as a colorless oil (178 mg, 62%). Applying the procedure above, the phenol (58 mg) was converted to the methylcarbamate, affording **4g** as a yellow oil (55 mg, 74% yield). ^1^H NMR (400 MHz, CDCl_3_) δ 7.08–6.98 (m, 2H), 6.87 (ddd, *J* = 9.6, 2.2, 2.2 Hz, 1H), 5.09 (br s, 1H), 2.93 (d, *J* = 4.0 Hz, 0.15H, minor carbamate rotamer), 2.87 (d, *J* = 4.0 Hz, 2.85Hz, major carbamate rotamer), 0.94 (t, *J* = 7.8 Hz, 3H), 0.70 (q, *J* = 7.8 Hz, 2H), 0.23 (s, 9H); ^13^C NMR (101 MHz, CDCl_3_) δ 162.5 (d, ^1^J_CF_ = 248.4 Hz), 154.8, 151.5 (d, ^2^J_CF_ = 9.7 Hz), 143.2 (d, ^3^J_CF_ = 4.6Hz), 121.8 (d, ^3^J_CF_ = 2.9 Hz), 116.6, 109.9 (d, ^2^J_CF_ = 24.2), 27.7, 7.23, 7.13, and −3.73.

2-(propylthio)phenyl methylcarbamate (**6a**) 2-mercaptophenol (300 mg, 2.5 mmol) was dissolved in dry DMF (5 mL) and NaHCO_3_ (630 mg, 3 equivalent) and propyl bromide (0.45 g, 5.0 equivalent) were added. After heating to 55 °C for 16 h, aqueous workup, and column chromatography, 2-propylthiophenol was isolated as a pale oil (391 mg, 98%). 2-propylthiophenol (354 mg, 2.11 mmol) was dissolved in dry THF (20 mL), treated with NaH (60% in mineral oil, 110 mg, 2.75 equivalent), and methylcarbamoyl chloride (394 mg, 4.2 equivalent) was added. Aqueous workup and column chromatography afforded **6a** as a pale oil (360 mg, 76%). ^1^H NMR (400 MHz, CDCl_3_) δ 7.33 (dd, *J* = 5.6, 1.8 Hz, 1H), 7.21–7.15 (m, 2H), 7.11 (dd, *J* = 5.3, 1.9 Hz, 1H), 5.08 (br s, 1H), 3.02 (d, *J* = 4.0 Hz, 0.15H, minor carbamate rotamer), 2.91 (d, *J* = 4.0 Hz, 2.85Hz, major carbamate rotamer), 2.85 (t, *J* = 5.8 Hz, 2H), 1.66 (hextet, *J* = 5.9 Hz, 2H), 1.02 (t, *J* = 5.9 Hz, 3H); ^13^C NMR (101 MHz, CDCl_3_) δ 154.8, 149.3, 130.5, 129.5, 126.7, 126.2, 123.1, 34.9, 28.0, 22.5, and 13.6.

3-(tert-butyl)phenyl methyl(phenylthio)carbamate (**7a**): Compound **1a** (150 mg) was treated with triethylamine (4 equiv) and benzenesulfenyl chloride (1.5 equvalent) in carbon tetrachloride (4 mL) at 45 °C for 18 h. Following aqueous workup the residue was chromatographed in 30:1 hexanes:ethyl acetate to afford **7a** as a yellow oil (186 mg, 82% yield). ^1^H NMR (400 MHz, CDCl_3_) δ 7.40–7.36 (m, 2H), 7.34 (d, *J* = 9.5 Hz, 2H), 7.30–7.22 (m, 3H), 7.08 (s, 1H), 6.92 (d, *J* = 8.2 Hz, 1H), 3.43 (s, 3H), 1.30 (s, 9H); ^13^C NMR (101 MHz, CDCl_3_) δ 156.8, 153.0, 151.3, 137.5, 129.3, 128.8, 127.3, 126.3, 125.3, 122.8, 118.5, 42.0, 34.8, and 32.2.

3-(tert-butyl)phenyl methyl(p-tolylthio)carbamate (**7b**): Following the procedure for **7a**, **1a**, and p-toluylsulfenyl chloride were reacted to afford **7b** as a yellow oil (213 mg, 89%). ^1^H NMR (400 MHz, CDCl_3_) δ 7.36–7.22 (m, 6H), 7.11 (s, 1H), 6.96 (br s, 1H), 3.42 (s, 3H), 2.38 (s, 3H), 1.32 (s, 9H); ^13^C NMR (101 MHz, CDCl_3_) δ 156.8, 154.9, 151.4, 138.2, 134.1, 130.0, 128.8, 127.2, 122.7, 118.6, 41.9, 34.8, 31.3, and 21.2.

3-(tert-butyl)phenyl ((4-(tert-butyl)phenyl)thio)(methyl)carbamate (**7c**): following the procedure for **7a**, **1a**, and 4-t-butylphenylsulfenyl chloride were reacted to afford **7c** as a yellow oil (152 mg (73%). ^1^H NMR (400 MHz, CDCl_3_) δ 7.43 (d, *J* = 9.7 Hz, 2H), 7.37 (d, *J* = 9.7 Hz, 2H), 7.30 (t, *J* = 8.0 Hz, 1H), 7.25 (d, 8.0 Hz, 1H), 7.10 (s, 1H), 6.95 (d, *J* = 8.0 Hz, 1H), 3.42 (s, 3H), 1.34 (s, 9H), 1.31 (s, 9H); ^13^C NMR (101 MHz, CDCl_3_) δ 156.8, 152.9, 151.4, 151.2, 134.3, 128.8, 126.9, 126.3, 122.7, 118.6, 41.9, 34.8, 34.7, 31.6, 31.3, 31.2, 22.7, and 14.1.

3-(trimethylsilyl)phenyl methyl(phenylthio)carbamate (**7d**): Following the procedure for **7a**, **3a** and benzenesulfenyl chloride were reacted to afford **7d** as a yellow oil (68 mg, 58%). ^1^H NMR (400 MHz, CDCl_3_) δ 7.44–7.30 (m, 6H), 7.28–7.23 (m, 1H), 7.19 (s, 1H), 7.09 (s, 1H), 3.47 (s, 3H), 0.27 (s, 9H); ^13^C NMR (101 MHz, CDCl_3_) δ 156.9, 151.2, 142.7, 133.7, 130.7, 129.4, 129.0, 127.5, 125.9, 122.0, 42.1, and −1.11.

4-methyl-3-(trimethylsilyl)phenyl methyl(phenylthio)carbamate (**7e**): Following the procedure for **7a**, **4c** and benzenesulfenyl chloride were reacted to afford **7e** as a yellow oil (166 mg, 58%). ^1^H NMR (400 MHz, CDCl_3_) δ 7.40 (d, *J* = 8.7 Hz, 2H), 7.39–7.35 (m, 2H), 7.29 (t, *J* = 8.7, Hz, 1H), 7.19–7.10 (m, 2H), 7.00 (d, *J* = 8.0 Hz, 1H), 3.44 (s, 3H), 2.44 (s, 3H), 0.27 (s, 9H); ^13^C NMR (101 MHz, CDCl_3_) δ 157.0, 149.0, 140.9, 140.1, 130.7, 129.3, 129.0, 127.3, 126.7, 125.6, 122.0, 42.0, 22.3, and −0.4.

### 2.2. Insects and Reagents

*Anopheles gambiae* (insecticide-susceptible G3 strain), were taken from colonies cultured in the Department of Entomology at Virginia Polytechnic Institute and State University, or the University of Florida, Emerging Pathogens Institute. Acetylthiocholine (ATChI), recombinant human enzyme (hAChE), 5,5’-dithio-*bis*-(2-nitrobenzoic acid) (DTNB), and all buffer components were purchased from Sigma–Aldrich (MO, USA). Whole mosquito homogenate was prepared essentially as described by Anderson et al. [11], and was used as the source of *Ag*AChE.

### 2.3. AChE Inhibition Assays

AChE enzyme inhibition assays were performed using the Ellman [12] method adapted for a 96-well microplate assay [11], with a few modifications. Briefly, inhibitor stocks of 0.01 M were freshly prepared in dimethylsulfoxide (DMSO) followed by serial dilutions in DMSO. A 100-fold dilution into sodium phosphate buffer (pH 7.8) was made for each of the DMSO dilutions. The final DMSO concentration was maintained at 0.1% v/v and concentrations of the drug typically ranged from 1 nM to 0.1 mM in 10-fold steps. After a 10 min pre-incubation of the enzyme and inhibitor, hydrolysis of the Ellman’s reagents (ATChI and DTNB) was monitored for 10 mins at 405 nm, in a Dynex 96-well plate reader (Dynex, Chantilly, VA, USA). Percent residual AChE activity values (relative to the control) were plotted in Prism^®^ (GraphPad, USA) and analyzed by non-linear regression (curve-fit) to generate IC_50_ values. Half maximal inhibitory concentration (IC_50_) is defined as the inhibitor concentration that blocks 50% of the enzyme activity and is used to define the potency of the inhibitor. Mosquito selectivity (S) was determined by the IC_50_ ratio of hAChE/*Ag*AChE for each compound. Statistical significance of both potency and toxicity of the compounds was assessed based on non-overlapping of 95% confidence intervals (95% CI).

### 2.4. Insect Bioassays

A standard WHO-treated filter paper assay [13] was used to assess contact toxicity of the structural analogs, dissolved in ethanol and without any added silicon or other oil, which reduced activity (data not shown). An initial range finding assay was performed with concentrations of 0.1, 0.5, and 1 mg/mL of each compound, to identify additional test concentrations needed to obtain an LC_50_ value. From this initial assay, only compounds showing greater than 50% mortality at 0.5 mg/mL were tested further, with up to five concentrations per compound and 25 mosquitoes (2–5-day old sugar-fed females) per treatment. Assays were repeated at least twice using different batches of mosquitoes to account for inter-batch variability. Treated mosquitoes and untreated controls were maintained at 75% RH and 25.6 °C. Mortality was assessed after 24 h and corrected for control mortality by the method of Abbott [14]. Data was plotted in Poloplus^®^ and a probit analysis performed to generate LC_50_ values; the carbamate concentration that killed 50% of the exposed mosquitoes. Statistical significance of different IC_50_ values was judged by non-overlap of the 95% confidence intervals.

## 3. Results

### 3.1. AChE Potency and Mosquito Toxicity of Terbam (***1a***) Analogs

We have previously disclosed results with terbam (3-*t*-butylphenyl methylcarbamate) **1a**. It displayed excellent potency for inhibiting *Ag*AChE and excellent contact toxicity to *An. gambiae* (LC_50_ = 37 µg/mL), but enzymatic inhibition selectivity (S) of only 12-fold [5]. Except for **2a**, all modifications using **1a** as a template involved holding the 3-*t*-butyl group constant. Bromination at the 4-position (**1b**) led to a 16-fold loss in *Ag*AChE inhibitory potency, compared to a 4-fold loss in hAChE inhibitory potency, relative to **1a** (Table 1). Bromination at the 6-position (**1d**) increased inhibitory potency to both AChEs (about 2-fold for *Ag*AChE and 7-fold for hAChE), but reduced toxicity to mosquitoes 7-fold (Table 1). A 6-iodo substituent (**1e**) led to 58-fold and 2-fold loss in potency to *Ag*AChE and hAChE, respectively, and a significant loss of mosquitocidal activity. Finally, addition of a 6-hexyl group (**1f**) drastically reduced *Ag*AChE inhibitory potency while only reducing hAChE inhibitory potency 20-fold (Table 1).

Installation of a 4-carboxamido group abrogated *Ag*AChE inhibition and mosquito toxicity (**1h**–**1l**). For hAChE, however, we observed micromolar IC_50_ values with the allyl (**1h**), *N-*di-ethyl (**1k**), and *N*-ethyl (**1l**) carbamoyl analogs, but no activity was observed with the methyl (**1i**) and iso-butyl (**1j**) analogs (Table 1). Addition of a thioether functionality at C6, as exemplified by compounds **1m–1o**, caused significant loss of *Ag*AChE inhibition potency; 27-fold with **1m** and much more with **1n** and **1o**, and they had little insecticidal activity. Finally, when the *tert*-butyl group of **1a** was replaced with the smaller and highly electron-withdrawing trifluoromethyl moiety (**2a**), inhibitory potency to both mosquito and human AChEs was lost, but surprisingly the compound was toxic to mosquitoes, although it was less toxic than **1a** by about 10-fold (Table 1).

### 3.2. SAR of 3-Trialkylsilyl- and 2-Thioalkyl-Substituted Methylcarbamates

**Figure 2:**
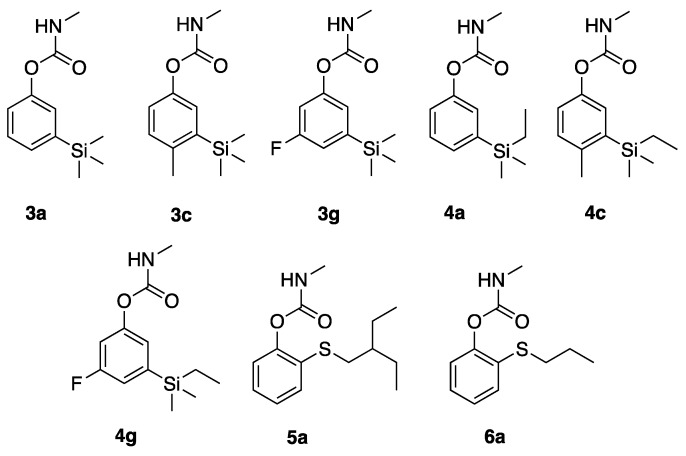
Analogs of 3-trialkylsilyl- and 2-thioalkyl-substituted methylcarbamates.

Close structural analogs of **1a** featured replacement of the *t*-butyl group with trialkylsilyl groups, and these analogs had similar inhibition potency and selectivity, but reduced contact toxicity as exemplified by **3a** and **4a**, which were reported in a previous study [5]. Thus, analogs of **3a** and **4a**, variants featuring 4-methyl (**3c**, **4c**) and 5-fluoro (**3g**, **4g**) were prepared (Figure 2) and assayed (Table 2). Both substitutions reduced *Ag*AChE inhibition potency, and 5-fluorination had a significantly more deleterious effect for the 3-SiMe_3_ derivative than for the 3-SiEtMe_2_ derivative (**3g** and **4g**). None of these variants had improved enzymatic selectivity, and reductions in *Ag*AChE inhibition potency correlated with reduced mosquitocidal action (Table 2).

One additional 2-thioalkylphenyl methylcarbamate (**6a**) (Figure 2) was also synthesized based upon previous studies that identified compound **5a** [5], as having good enzyme selectivity (135-fold), but poor contact toxicity on paper (Table 2). As can be seen, 2-thiopropylphenyl methylcarbamate **6a** had a 3-fold reduction of inhibitory potency to *Ag*AChE, and 7-fold reduced inhibition selectivity, but had improved paper toxicity to adult *An. gambiae* (Table 2).

### 3.3. Toxicity of N-Sulfenylated Methylcarbamates ***7a***–***e***

**Figure 3:**
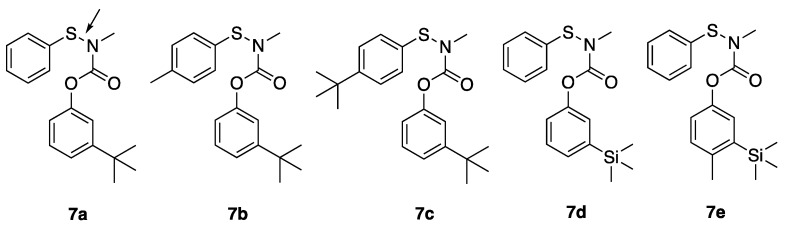
*N*-sulfenylated pro-insecticidal analogs of 3-*t*-butyl-phenyl- and 3-trimethylsilyl-phenyl methylcarbamates.

*N*-Sulfenylated methylcarbamates (Figure 3) were also tested because these pro-insecticides have been shown to have lower mammalian toxicity; hydrolysis of the labile sulfur-nitrogen bond in vivo yields the active insecticide [15]. *N*-Sulfenylated analogs of **1a**, **3a**, and **3c** were synthesized using thiophenyl or *para*-substituted thiophenyl groups (**7a**–**7e)**. Among the *N*-sulfenylated compounds, only **7a** and **7b** showed contact toxicity to mosquitoes in the WHO paper assay. It was observed that **7a** killed 70% of G3 mosquitoes in 24 h at 0.5 mg/mL but was not toxic at 0.1 mg/mL. Likewise, **7b** had 100% G3 mortality in 24 h at 1 mg/mL but was not toxic at 0.5 mg/mL. Despite showing modest paper contact toxicity, **7a** was highly toxic to G3 mosquitoes when topically applied, with an LD_50_ = 2.0 ng/female and 95% CI of 1.8–2.3.

## 4. Discussion

This study initiated a synthesis and testing program with the goal of expanding the existing SAR as it pertains to carbamate inhibition of *Ag*AChE and hAChE, as well as toxicity. Due to the overall limited number of analogs made for each chemical series, this work constitutes a search for new leads as opposed to a thorough exploration of SAR of these two enzymes. It is noteworthy that much of the classical literature on carbamates focused on activity against housefly AChE and topical toxicity to this species, as well as larval mosquito bioassays, which makes comparisons to the data of the present study somewhat difficult.

### 4.1. Effects of Structural Modification of ***1a***: Implications for the AgAChE Active Site

The AChE–ligand interactions and molecular docking experiments with structurally diverse carbamates have been extensively explored in previous studies [4,5,11]. Examining the data in Table 1, it is clear that *Ag*AChE can accommodate only relatively small substituents at the 6-position of terbam (**1a**). A 6-bromo-substituent (**1d**) is favorable, but the slightly larger 6-iodo (**1e**) and 6-thiomethyl (**1m**) substituents adversely impact *Ag*AChE inhibitory potency. The larger 6-hexyl (**1f**), 6-isobutylthio (**1n**), and 6-isopropylthio (**1o**) groups all have significantly reduced potency for inhibition of *Ag*AChE. Interestingly, hAChE inhibitory potency is less sensitive to steric bulk at the 6-position. In previous studies, SAR of 3-substituted phenyl carbamates suggested that *Ag*AChE had a slightly larger ligand binding pocket than hAChE in order to accommodate a 3-*t*-butyl group [5]; the present study suggests that in the vicinity of the 6-position, hAChE is more accommodating to steric bulk than *Ag*AChE. The design of the 4-carboxamido analogs **1h**–**l** was inspired by preliminary molecular modeling (data not shown) that suggested a hydrogen-bond acceptor at the C4 position of **1a** might increase inhibition potency. Unfortunately, these compounds were even less potent inhibitors of *Ag*AChE than the 4-bromo analog **1b**. The last analog of **1a** explored was **2a**, which replaced the 3-*t*-Bu with 3-CF_3_. We propose that the drastic decrease in inhibition potency can be attributed to electronic effects. Sterically, the CF_3_ group was approximately intermediate in size compared to the isopropyl and *t*-butyl groups [16], so there should be plenty of space for it in the AChE active site. Thus, the poor inhibition potency of **2a** may be due to repulsive interaction of the electron-rich CF_3_ group with one or more of the aromatic amino acid sidechains that are present in the active site of *Ag*AChE [4]. Reviewing the hAChE potency of these inhibitors in Table 1, none of these modifications improved enzymatic selectivity, and in many cases reversed the selectivity to be more potent against hAChE, which was especially true of the carbamoylated phenyl ring analogs, **1h**–**l**. Lastly, in general, the decreases seen in *Ag*AChE inhibition potency were reflected by decreased *An. gambiae* contact toxicity. Interestingly, however, compound **2a** exhibited only a 10-fold decrease in contact toxicity relative to **1a**, despite its drastically reduced (>300-fold) *Ag*AChE inhibition potency. A convincing explanation for this discrepancy remains elusive at present.

The AChE–ligand interactions and molecular docking experiments with structurally diverse carbamates have been extensively explored in previous studies [4,5,11]. Examining the data in Table 1, it is clear that *Ag*AChE can accommodate only relatively small substituents at the 6-position of terbam (**1a**). A 6-bromo-substituent (**1d**) is favorable, but the slightly larger 6-iodo (**1e**) and 6-thiomethyl (**1m**) substituents adversely impact *Ag*AChE inhibitory potency. The larger 6-hexyl (**1f**), 6-isobutylthio (**1n**), and 6-isopropylthio (**1o**) groups all have significantly reduced potency for inhibition of *Ag*AChE. Interestingly, hAChE inhibitory potency is less sensitive to steric bulk at the 6-position. In previous studies, SAR of 3-substituted phenyl carbamates suggested that *Ag*AChE had a slightly larger ligand binding pocket than hAChE in order to accommodate a 3-*t*-butyl group [5]; the present study suggests that in the vicinity of the 6-position, hAChE is more accommodating to steric bulk than *Ag*AChE. The design of the 4-carboxamido analogs **1h**–l was inspired by preliminary molecular modeling (data not shown) that suggested a hydrogen-bond acceptor at the C4 position of **1a** might increase inhibition potency. Unfortunately, these compounds were even less potent inhibitors of *Ag*AChE than the 4-bromo analog **1b**. The last analog of **1a** explored was **2a**, which replaced the 3-*t*-Bu with 3-CF_3_. We propose that the drastic decrease in inhibition potency can be attributed to electronic effects. Sterically, the CF_3_ group was approximately intermediate in size compared to the isopropyl and *t*-butyl groups [16], so there should be plenty of space for it in the AChE active site. Thus, the poor inhibition potency of **2a** may be due to repulsive interaction of the electron-rich CF_3_ group with one or more of the aromatic amino acid sidechains that are present in the active site of *Ag*AChE [4]. Reviewing the hAChE potency of these inhibitors in Table 1, none of these modifications improved enzymatic selectivity, and in many cases reversed the selectivity to be more potent against hAChE, which was especially true of the carbamoylated phenyl ring analogs, **1h**–**l**. Lastly, in general, the decreases seen in *Ag*AChE inhibition potency were reflected by decreased *An. gambiae* contact toxicity. Interestingly, however, compound **2a** exhibited only a 10-fold decrease in contact toxicity relative to **1a**, despite its drastically reduced (>300-fold) *Ag*AChE inhibition potency. A convincing explanation for this discrepancy remains elusive at present.

### 4.2. Structural Modification of Trialkylsilylphenyl Methylcarbamate and 2-Thioalkyl Methylcarbamates

Turning to analogs of the **3a** and **4a**, variants featuring 4-methyl (**3c**, **4c**) and 5-fluoro (**3g**, **4g**) were prepared and assayed. Both substitutions reduced *Ag*AChE inhibition potency, notably, 5-fluorination had a significantly more deleterious effect for the 3-SiMe_3_ derivative than for the 3-SiEtMe_2_ derivative (**3g** and **4g**). None of these variants had improved enzymatic selectivity, and reductions in *Ag*AChE inhibition potency correlated with reduced mosquitocidal action.

A single variant of 2-thioalkyl-substituted **5a** featured a smaller alkyl group (propyl, **6a**). This compound proved less potent and selective for inhibition of *Ag*AChE, relative to **5a**. Interestingly, the insecticidal potency of **6a** improved relative to **5a**, which may reflect reduced oxidative metabolism in the mosquito or better transfer off of paper. In a previous study of *ortho* thioalkyl phenylcarbamates, compound **6a** had essentially optimal activity in this series for both housefly AChE inhibition and *Culex pipiens* larval toxicity [3]. Moreover, the addition of piperonyl butoxide to housefly toxicity bioassays increased toxicity, indicating that sulfur oxidation was not bioactivating as it is for aldicarb [3]; on the contrary, these results suggest oxidative detoxication.

### 4.3. Paper versus Topical Toxicity of N-Sulfenylated N-Methylcarbamates ***7a***–***e***

*N*-arylsulfenyl and *N*-alkylsulfenyl derivatives of methylcarbamate insecticides have been shown to possess lower mammalian toxicity and be more effective mosquito larvicides than the parent methylcarbamates [15]. This same study also reported that the selective toxicity did not relate directly to anticholinesterase activity. Thus, selectivity of these compounds was likely due to differential metabolism. Toxicity of arylsulfenylated compounds to mice was lowered up to 50-fold in these compounds, but was 5- to 17-fold lower for alkylsulfenylated analogs [15]. With such confirmed improvement of mammalian safety, as well as increased toxicity to insects, a new generation of sulfenylated compounds was synthesized for testing. However, sulfenylated analogs of **1a**, **3a**, and **3c** did not show good paper contact toxicity to mosquitoes, but **7a** was twice as toxic to G3 mosquitoes topically, compared to **1a**, its non-sulfenylated parent. The topical toxicity of the other compounds has not been determined, and the lack of paper contact toxicity of **7a**, even though it is highly toxic when topically applied, cannot be fully addressed at this point

## 5. Conclusions

Structural modifications of promising anticholinesterase mosquitocides **1a**, **3a**, **4a**, and **5a** were examined in this paper. A series of 6-substituted analogs of **1a** analogs indicated *Ag*AChE cannot accommodate a substituent larger than bromine in this position. A group of 4-substituted analogs of **1a**, **3a**, and **4** indicates that *Ag*AChE cannot accommodate any of the attempted substitutions in this position. Replacement of the *t*-butyl group of **1a** with CF_3_ resulted in poor anticholinesterase activity, but surprisingly this compound (**2a**) did have measurable mosquitocidal activity. The 2-thiopropyl analog of **5a** (**6a**) possessed lower *Ag*AChE inhibition potency but improved toxicity to adult mosquitoes. Finally, *N*-sulfenylated proinsecticidal derivatives of **1a** and **3a** that were synthesized had poor contact activity on paper, but one of them (**7a**) had good topical activity.

## Figures and Tables

**Figure 1:**
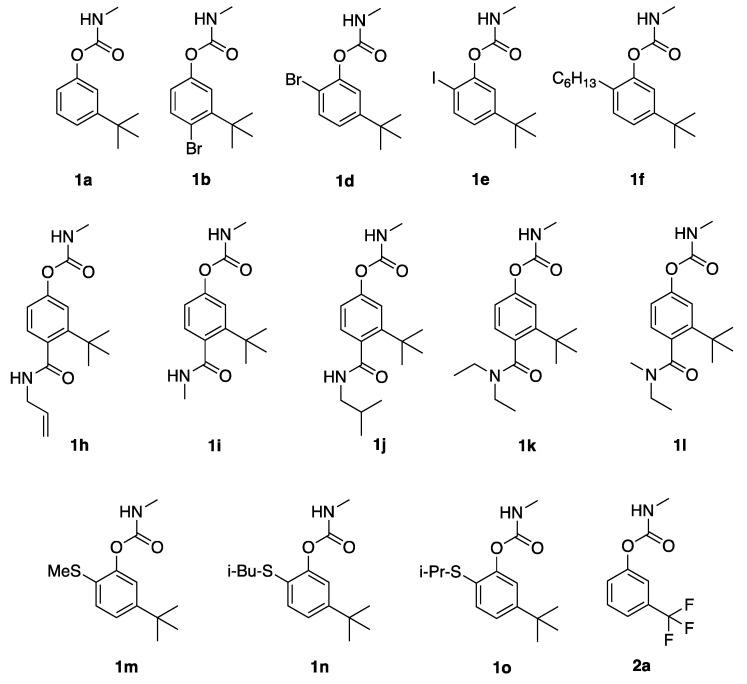
Terbam (**1a**) and structural analogs described in this study.

**Table 1:** AChE inhibition and mosquito toxicity to adult female *An. gambiae* by 3-*tert*-butylphenyl carbamate **1a** and the substituted analogs shown in Figure 1.

Compound	*Ag*AChE, IC50, ^a^ nM	hAChE ^a^IC50, nM	S ^b^	LC_50_ or % Mortality ^c^
**1a** ^d^	36 (34–38) ^a^	* 320 (293-349) ^a^	9	37 (14–60) ^a^
**1b**	580 (260–892) ^b^	* 1200 (913–1533) ^b^	2	600 (570–627) ^b^
**1d**	14 (12–17) ^c^	* 48 (40–56) ^c^	3.4	260 (239–276) ^c^
**1e**	2100 (1195–2309) ^d^	* 640 (538–752) ^d^	0.3	20%
**1f**	>10^5^	6300 (5126–7460) ^e^	<0.06	4%
**1h**	>10^5^	2800 (1719–4677) ^f,g^	<0.03	0%
**1i**	>10^5^	>10^5^	-	4%
**1j**	>10^5^	>10^5^	-	0%
**1k**	>10^5^	2300 (1189–4448) ^b,f.g^	<0.023	0%
**1l**	>10^5^	5500 (2830–8128) ^e,f,g^	<0.06	0%
**1m**	970 (830–1172) ^b^	* 640 (572–719) ^d^	0.66	0%
**1n**	32,000 (23,060–63,300) ^e^	* 4700 (3777–5784) ^e,f^	0.15	4%
**1o**	75,000 (N/A)	2200 (1653–2941) ^g^	0.03	16%
**2a**	>10^5^	>10^5^	-	390 (333–471) ^d^

^a^ IC_50_ with (95% CI); ^b^ S = selectivity ratio (IC_50_ hAChE/*Ag*AChE), and and asterisk indicates an IC_50_ value for hAChE that is significantly different from *Ag*AChE, as judged by non-overlap of the 95% CI; ^c^ LC_50_ in µg/ml or % mortality at 1 mg/mL; IC_50_s or LC_50_s within a column not labeled by the same lower case letter are significantly different (*p* > 0.05), as judged by non-overlap of the 95% CI.

**Table 2:** Enzyme inhibition potency and toxicity to adult female *An. gambiae* of chemical structures shown in Figure 2.

Compound	*Ag*AChE IC_50_^a^ nM	hAChE IC_50_^a^, nM	S ^b^	LC_50_^c^ or % Mortality ^c^
**3a**	50 (40–64) ^a^	* 490 (452–526) ^a^	9.8	170 (162–176) ^a^
**3c**	380 (318–450) ^b^	* 2400 (2032–2814) ^b^	6.3	240 (154–330) ^ac^
**3g**	2600 (1260–5453) ^c^	4200 (3714–4616) ^c^	1.6	4%
**4a**	72 (67–78) ^d^	* 630 (515–761) ^a,d^	8.8	190 (154–229) ^a^
**4c**	630 (556–714) ^e^	* 1400 (1205–1559) ^e^	2.2	16%
**4g**	190 (158–225) ^f^	* 1900 (1649–2245) ^f^	10	440 (405–474) ^b^
**5a**	37 (32–43) ^a^	* 5000 (4514–5618) ^g^	135	27%
**6a**	110 (135–152) ^g^	* 2300 (2019–2596) ^f^	21	340 (321–361) ^c^

^a^ IC_50_ with (95% CI); ^b^ S = selectivity ratio (IC_50_ hAChE / *Ag*AChE), and and asterisk indicates an IC_50_ value for hAChE that is significantly different from *Ag*AChE, as judged by non-overlap of the 95% CI; ^c^ LC_50_ in µg/mL or % mortality at 1 mg/mL; IC_50_s or LC_50_s within a column not labeled by the same lower case letter are significantly different (*p* > 0.05), as judged by non-overlap of the 95% CI.
